# Do Rare Stimuli Evoke Large P3s by Being Unexpected? A Comparison of Oddball Effects Between Standard-Oddball and Prediction-Oddball Tasks

**DOI:** 10.5709/acp-0189-9

**Published:** 2016-06-30

**Authors:** Rolf Verleger, Kamila Śmigasiewicz

**Affiliations:** 1 Department of Neurology, University of Lübeck, Germany; 2 Institute of Psychology II , University of Lübeck, Germany

**Keywords:** P3b, expectancies, oddball, prediction

## Abstract

The P3 component of event-related potentials increases when stimuli are rarely presented. It has been assumed that this *oddball effect* (rare-frequent difference) reflects the unexpectedness of rare stimuli. The assumption of unexpectedness and its link to P3 amplitude were tested here. A standard- oddball task requiring alternative key-press responses to frequent and rare stimuli was compared with an *oddball-prediction* task where stimuli had to be first predicted and then confirmed by key-pressing. Oddball effects in the prediction task depended on whether the frequent or the rare stimulus had been predicted. Oddball effects on P3 amplitudes and error rates in the standard oddball task closely resembled effects after *frequent* predictions. This corroborates the notion that these effects occur because frequent stimuli are expected and rare stimuli are unexpected. However, a closer look at the prediction task put this notion into doubt because the modifications of oddball effects on P3 by expectancies were entirely due to effects on frequent stimuli, whereas the large P3 amplitudes evoked by rare stimuli were insensitive to predictions (unlike response times and error rates). Therefore, rare stimuli cannot be said to evoke large P3 amplitudes because they are unexpected. We discuss these diverging effects of frequency and expectancy, as well as general differences between tasks, with respect to concepts and hypotheses about P3b’s function and conclude that each discussed concept or hypothesis encounters some problems, with a conception in terms of subjective relevance assigned to stimuli offering the most consistent account of these basic effects.

## Introduction

 Event-related potentials (ERPs) are voltage changes recorded from the scalp in temporal coincidence to events (Luck & Kappenman, 2012). When some task has to be performed with these events, a prominent ERP component is the large positive potential, termed P3 (Ritter, Vaughan, & Costa, 1968) or P300 ( Donchin & Cohen, 1969). Usually, the P3 complex consists of two components: the fronto-centrally recorded P3a, related to stimulus novelty, and the larger parietally recorded P3b (Dien, Spencer, & Donchin, 2004; Gaeta, Friedman, & Hunt, 2003; Polich, 2007; Squires, Squires, & Hillyard, 1975; Verleger, Jaśkowski, & Wauschkuhn, 1994). 

 A main characteristic of P3 is that it is larger after rarely than after frequently occurring stimuli when two stimuli are presented in unpredictable series (oddball effect: Duncan-Johnson & Donchin, 1977; Squires et al., 1975). This has usually been attributed to participants’ subjective impression of “unexpectedness”, as succinctly summarized by Johnson (1986) . Thus, it has been assumed that: (i) rare stimuli are unexpected, (ii) P3 is increased when stimuli are unexpected, and (iii) this increase by unexpectedness accounts for the effect of frequency. In line with assumption (i) are hundreds of studies showing that behavioral responses are slower with rare than with frequent stimuli (e.g., Miller, 1998). In line with assumption (ii) larger P3s were evoked by unpredicted stimuli when participants made explicit predictions about which of two equiprobable stimuli would be presented (Matt, Leuthold, & Sommer, 1992; Munson, Ruchkin, Ritter, Sutton, & Squires, 1984; Sutton, Braren, Zubin, & John, 1965; cf. Ritter, Sussman, Deacon, Cowan, & Vaughan, 1999). However, these effects were not large, nor did they provide evidence on assumption (iii) because the alternative stimuli were equally frequent rather than frequent and rare. Indeed, all three assumptions can be contested. With regard to assumption (i) it has been argued that rare stimuli are simultaneously unexpected and expected (“awaited”, Verleger, 1988): unexpected by probability of occurrence but awaited by their relevance, being perceived by participants and experimenters as the stimuli which the task is about (Bouret & Sara, 2005; Verleger, 1988). With regard to assumption (ii) in the few studies in which P3 amplitudes evoked by rare stimuli were compared between predictable and unpredictable situations, centro-parietal P3b amplitudes were equally large for predictable and unpredictable stimuli (Fogelson, Shah, Bonnet-Brilhault, & Knight, 2010; Fogelson et al., 2009; Verleger et al., 1994) casting doubts on assumption (ii). In this line, in a series of studies Sommer and colleagues compared effects of explicit subjective expectancies about the next stimulus with effects of preceding objective sequences of alternating or repeating stimuli (Matt et al., 1992; Sommer, Matt, & Leuthold, 1990; Sommer, Leuthold, & Soetens, 1999) and found that P3 amplitudes were more affected by objective sequences than by explicit expectancies (Sommer, Leuthold, & Matt, 1998). Those authors put forward Kahneman and Tversky’s (1982) suggestion that P3 reflects violations of passive primed dispositions rather than of active, conscious expectancies. Such passive priming would also allow for two expectations occurring simultaneously: Responses to frequent and rare stimuli might be primed in parallel (Sommer et al., 1999). To summarize, it is still unclear whether P3b is larger with unexpected than expected stimuli (assumption ii) and it remains unclear whether any increase of P3 by unexpectedness can account for the effect of frequency on P3 (assumption iii). 

 This sole dependence on passive dispositions primed by preceding stimuli seems somewhat implausible in light of the well-known large impact of higher-level factors on P3, like task relevance and informational value of stimuli (Pitts, Metzler, & Hillyard, 2014; Squires et al., 1975; Sutton et al., 1965). A relevant point may be that stimulus alternatives were equally probable in those studies that tested the effects of subjective expectancies versus objective sequences (Matt et al., 1992 ; Munson et al., 1984; Sommer et al., 1990, 1999). Thus, whatever participants explicitly predicted did not have much basis in objective reality and might, therefore, have not been sufficiently strongly expected by participants to be reflected in P3. Therefore, in the present study the two stimuli to be predicted occurred with different frequencies. In order to elucidate the relations between expectancies and P3, P3 amplitudes, response times (RTs) and error rates were compared between a *standard oddball* choice-response task and an *oddball-prediction* task. In both tasks, one key had to be pressed to the frequent and another key to the rare stimulus. The major difference between tasks was that in the prediction task one of those two keys had to be additionally pressed in advance, to indicate the participant’s prediction about which stimulus would appear. Correct predictions were rewarded. The following hypotheses were stated. 

(1) Trivially, there will be frequency effects on P3, RTs, and error rates in both tasks: P3 will be larger, RTs will be slower, and error rates will be higher with rare than with frequent stimuli. The term *oddball effect* will be used for these effects of frequency, both on P3 and on the behavioral measures (RTs and error rates). Assessing the oddball effects on behavior will help in interpreting these effects on P3. The following hypotheses, therefore, refer to P3, RT, and errors, but this is not meant to imply that oddball effects will be the same on these three measures.

 (2) In the oddball-prediction task, oddball effects will depend on participant’s prediction (*frequent* or *rare*): Oddball effects will be smaller after rare than after *frequent* predictions (Verleger, Asanowicz, Werner, & Śmigasiewicz, 2015) because when having made *rare* predictions participants will be prepared to perceive and process rare stimuli. 

(3) Comparing these results with the standard oddball task will provide clues about expectancies held in this task and, therefore, about whether the large P3 evoked by rare stimuli in this task is evoked for the reason that rare stimuli are unexpected. To detail, the oddball effects in the standard-oddball task might be more similar to either *frequent* or *rare* predictions in the oddball-prediction task or might lie in-between. Thus:

(3.a) Oddball effects in the standard oddball task might be similar to effects after *frequent* predictions in the oddball-prediction task. This would indicate that it is the frequent stimuli that are expected in the standard oddball task and, therefore, suggest that the large P3 evoked by rare stimuli in this task is related to unexpectedness of these stimuli.

(3.b) Conversely, oddball effects in the standard oddball task might be similar to effects after *rare* predictions in the oddball-prediction task. This would indicate that it is the rare stimuli that are expected in the standard oddball task and, therefore, suggest that the large P3 evoked by rare stimuli in this task is related to expectedness of these stimuli.

(3.c) As a third alternative, oddball effects in the standard oddball task might lie in-between effects after *rare* and after *frequent* predictions in the oddball-prediction task. This would indicate that expectations, or primed dispositions, for both frequent and rare stimuli, are simultaneously active in the standard oddball task to varying degrees.

 (4) Apart from these effects on rare-frequent differences, there might be global differences between tasks, for example, by generally longer RTs or higher error rates or larger P3 amplitudes in the oddball-prediction than in the standard-oddball task. Such effects might reflect the presence of conscious predictions in the prediction task and their absence in the oddball task. Such global task differences have been already investigated in pioneering studies on the P3 (Donchin, Kubovy, Kutas, Johnson, & Herning, 1973; Donchin, Tueting, Ritter, Kutas, & Heffley, 1975). However, at those times there were technical restrictions on number of participants, number of recording sites, and graphical presentation of results. Moreover, those classical studies (as well as Johnson, 1986) did not present frequent and rare stimuli for this comparison but two equiprobable ones. Therefore, more data are still needed to better understand possible task differences. 

 The requirement of confirming the actually presented stimuli by overt key-press responses is unusual in prediction tasks (Sutton et al., 1965, and many others; though see Verleger & Cohen, 1978). To control for effects introduced by this requirement, another condition of the prediction task was included where participants did not have to make these overt confirmations. 

## Experiment

### Method

#### Participants

Twenty university students participated (15 females and 5 males, *M*_age_ = 24 years, range 20-37). All participants reported normal or corrected-to-normal vision and no history of neurological disorders. Informed written consent was obtained before the experiment, and participants were paid 15 17 € after the experiment depending on their success in predicting stimuli. Two more students had participated, but one was excluded from analysis due to failure to follow instructions and the other due to too many electroencephalography (EEG) artifacts.

#### Stimuli and Procedure

Participants were seated in a comfortable armchair in a darkened room, at about 1.1 m viewing distance from the 17’’ computer screen, and held a computer keyboard on their lap. In all conditions, the black letters *X* or *U*, randomly chosen, were presented on a light-grey screen for 200 ms in Helvetica 35-point font, with *X* presented in 80% and *U* in 20% of trials. Responses were made by pressing the left or right control key. Presentation® software 14.0 (www.neurobs.com) was used to present stimuli, register responses, and send stimulus and response codes to another computer which stored these codes with the recorded EEG.

**Figure 1.**
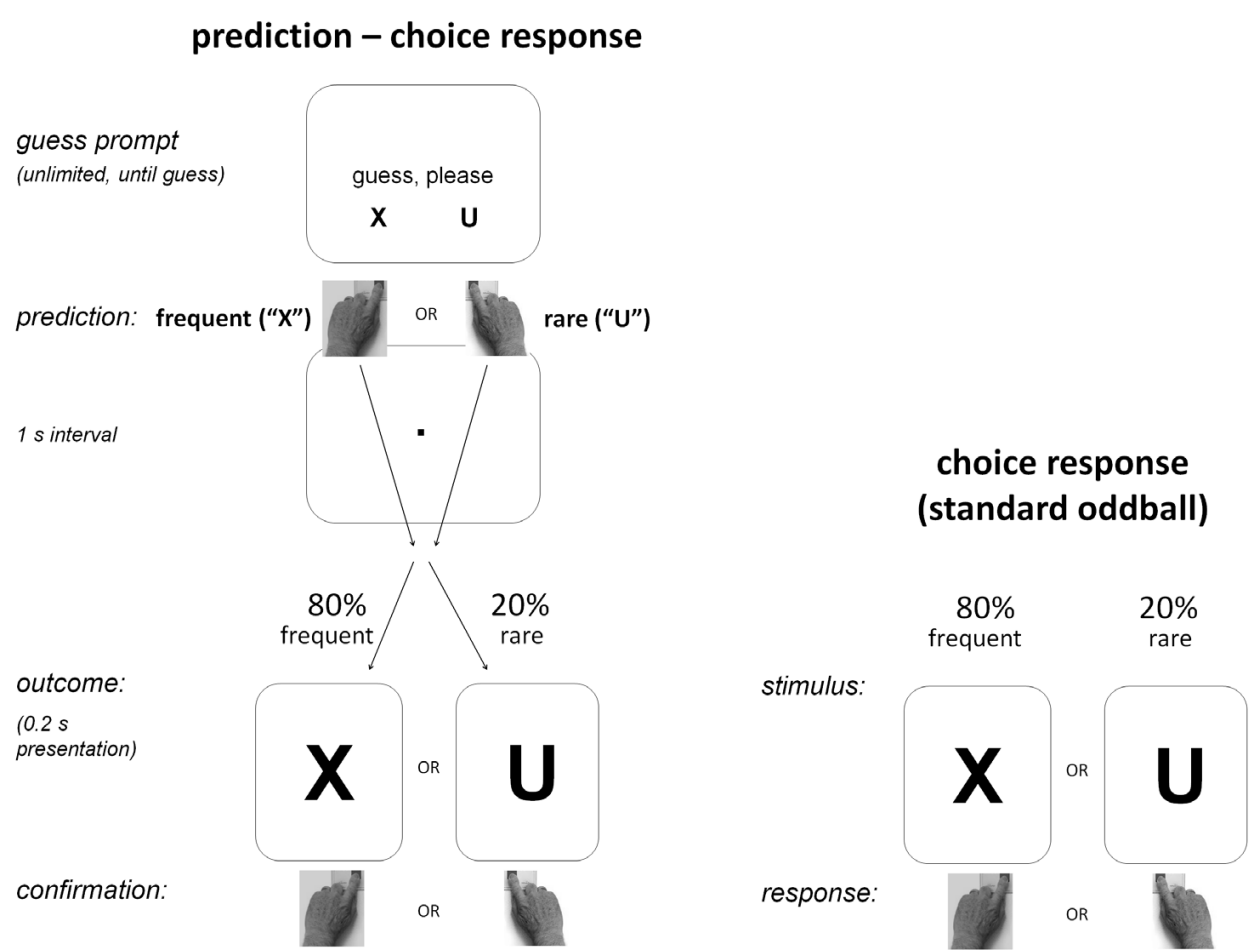
Outline of the paradigm. Both conditions were oddball choice-response tasks: Different keys had to be pressed in response to the frequent *X* and the rare *U*. In the prediction-C condition, participants had to predict the letter by pressing the appropriate key. Not depicted here is the prediction-noC condition where no confirmation was required in response to the predicted letter.

In the standard oddball task (right side of Figure 1) trials simply consisted of presenting the frequent X or the rare U, with participants having to press the appropriate key in response. The next stimulus was presented 0.9 s after the correct key was pressed. The task consisted of 250 trials. Assignment of left and right keys to the frequent *X* and rare *U* varied between participants.

In the oddball-prediction task, participants were informed by instruction presented on the screen that the task was a gamble requiring some luck, that they had to guess which of the two letters would occur, and that *X* would occur frequently and *U* rarely. Accurate guesses would yield 2 cents for frequent letters and 8 cents for rare letters. No money would be lost after inaccurate guesses. As illustrated in Figure 1, trials started with a prompt below screen center (“guess, please” in German, in black 20 pt. font) displaying the two black letters left and right below the prompt, as a reminder about which letter was assigned to which key. To prevent premature mechanical key-pressing, an error message (“pressed too early”, in German) appeared in large red 30 pt. font for 4 s whenever the keys were pressed before onset of the prompt. Key-pressing blanked the screen and was followed after 1 s by the frequent *X* or rare *U*. After every 20 trials and at block ending, summary feedback was given, separately for frequent and rare outcomes, on the number of correct guesses and the amount of money earned. The task consisted of 500 trials (twice as much as the standard oddball because trials had to be split for analysis by the prediction made before the stimuli). Assignment of left and right keys to the frequent and rare letters was constant for 250 trials, as in the standard oddball task, and then reversed, explicitly announced, in the middle of the task after a short break.

There were two versions of this task, differing in whether the outcome letter did or did not have to be confirmed (C) by appropriate key-press. Analysis will focus on the “prediction-C” task where this confirmation was required. In this task, letters were black as in the standard oddball task, and participants had to confirm the outcome letter by pressing the appropriate key, which was either the same key as used for predicting or (in case of incorrect predictions) the alternative key. The guess prompt of the next trial was presented 1 s after this confirming response. In the “prediction-noC” task, the condition without key-press confirmation, correctly and incorrectly predicted letters were presented in blue and yellow, respectively (colors balanced across participants) providing explicit feedback about guess accuracy (like in the standard condition of Verleger, Asanowicz, et al., 2015). The guess prompt of the next trial appeared 1.35 s after letter onset. To prevent carry-over from prediction-C, error messages (“do not press”, in German) appeared in large red 30 pt. font for 4 s in prediction-noC whenever the keys were pressed in response to the letters.

To avoid irrelevant effects of order, prediction-C either came first or last (vice versa prediction-noC) and *X* (the frequent stimulus) was first assigned either to the left or to the right key (vice versa the infrequent *U*). The standard oddball task was always in the middle, thus either before or after the prediction-C condition.

By necessity, the prediction and standard oddball tasks differed in the duration of the interval between two letters. Letter onsets were about 1.2 s apart in the oddball task (340 ms mean RT plus 900 ms response-stimulus interval) but about 2.9 s in the prediction task (400 ms mean RT plus 1 s from response to the next guess prompt, plus about 500 ms for making the prediction plus 1 s guess-stimulus interval). What was similar between tasks was the distance of letters from their preceding events which was the previous stimulus in the standard oddball and the guess prompt in the prediction task. This feature will be reconsidered in the Discussion.

Another possibly relevant difference was the reward associated to correctly predicted stimuli of the prediction task, kept in participants’ minds by the feedback screens provided after every 20 trials. We considered this necessary to motivate participants for remaining involved in making predictions rather than just mechanically pressing some key.

#### Analysis of behavior

Mean RTs of correct immediate (< 1200 ms) responses (confirmations in prediction-C and responses in the standard oddball task) as well as error rates (percentages of incorrectly responded trials) were submitted to analyses of variance (ANOVAs) on repeated measurements. First, an omnibus ANOVA was conducted on the standard oddball and the prediction-C tasks, with two levels of the factor Event Frequency (frequent, rare) and three levels of the factor Task & Prediction: standard oddball, *frequent* predictions, *rare* predictions. When significant, effects of Task & Prediction were elucidated in two ways: First, testing hypothesis (2) (cf. Introduction) by restricting analysis to the prediction-C task and, second, testing hypotheses (3) and (4) by comparing the standard oddball with the prediction-C task separately for frequent and *rare* predictions (in two ANOVAs with the factors Task and Event Frequency). Besides, in the prediction-C task, the percentages of trials in which participants predicted rare events were compared to the percentages of trials in which rare events actually occurred, in an ANOVA with the factor Subjective/Objective (prediction vs. actual frequency).

#### EEG recording and analysis

EEG was recorded with Ag/AgCl electrodes (Easycap, www.easycap.de) from 60 scalp sites, including eight midline positions from AFz to Oz and 26 pairs of symmetric left and right sites. Results from midline positions only will be reported. Additional electrodes were placed at the nose-tip for off-line reference and at Fpz as connection to ground. On-line reference was Fz. For artifact control, electrooculogram (EOG) was recorded, vertically (vEOG) from above versus below the right eye, and horizontally (hEOG) from positions next to the left and right tails of the eyes. Voltages were amplified from DC to 250 Hz by a BrainAmp MR plus, A-D converted, and stored at 500 Hz per channel. Off-line processing was done with Brain-Vision Analyzer software (version 2.03). Data were re-referenced to the nose-tip, low-pass filtered at 25 Hz, and segmented to epochs from 100 ms before to 1 s after letter onset. Epochs were rejected as gross artifacts when consecutive data points differed by more than 50 µV (except EOG and AF3, AFz, AF4, lest trials would be rejected for blinks). Then, ocular artifacts were corrected by using the linear regression method implemented in the Analyzer software. Next, data were referred to the mean amplitude of the first 100 ms as baseline in each channel, and trials were rejected when voltages exceeded ±150 µV in any EEG channel or when the wrong key was pressed. On average, 188 frequent and 30 rare trials remained for analysis in the standard oddball (minima 143 and 15), 303 and 55 after *frequent* predictions in prediction-C (minima: 236 and 24), and 75 and 16 after *rare* predictions (minima 20 and 8). Corresponding numbers for prediction-noC were 290, 70, 92, and 22 (minima 176, 43, 49, 10). EEG data were then averaged over trials, separately for the four guess-outcome combinations in the prediction tasks and for frequent and rare stimuli in the standard oddball task. Parameters were measured in these averaged waveforms. After inspecting these waveforms and their topographic distributions, the P3 complex was assessed to consist of the P3 peak and of the overlapping and following slow wave (SW). The main ANOVA was conducted on the P3 peak epoch which could be conveniently measured as mean amplitudes of the 300-500 ms epoch after letter onset at the seven midline sites Fz, FCz, Cz, CPz, Pz, POz, Oz. This ANOVA had the repeated-measurements factor Recording Site (7 levels) in addition to the factors used for analysis of behavior, Task/Prediction (standard oddball, *frequent*, *rare*) and Event Frequency (frequent, rare). Like with analysis of behavior, effects of Task/Prediction were further explored by separately analyzing the prediction-C task and by comparing the standard oddball with prediction-C separately after *frequent* and after *rare* predictions. To evaluate the impact of the SW, the P3 complex was also quantified as mean amplitude 300-700 ms after letter onset. These amplitudes were compared with the 300-500 ms amplitudes in an ANOVA with the additional factor Measurement Epoch.

To clarify interactions, ANOVAs were conducted on the single levels of the interacting factors. Degrees of freedom of the Task/Prediction and Recording Site factors were corrected with the Greenhouse-Geisser method. Corrected p-values will be reported whereas ε values will not be indicated, for brevity. Likewise, partial eta-squared will not be explicitly indicated, being easily derived from the reported F-values by the formula η_p_^2^ = (*F*/*df*)/(1+*F*/*df*).

## Results

### Behavior

#### Error rates and Response Times

**Figure 2.**
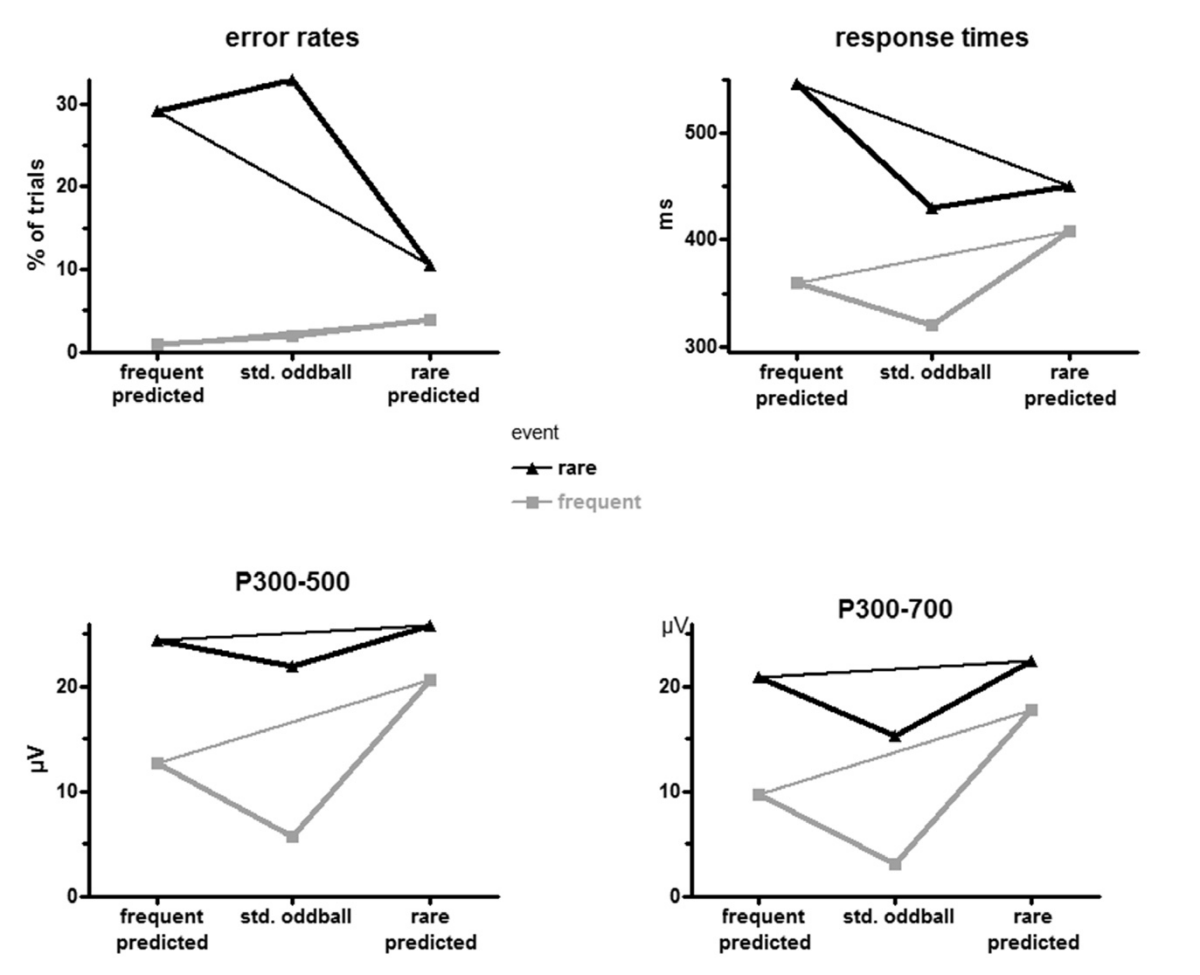
Overview of results. Behavioral results (error rates and response times) are at the top, measures of the P3 complex (recorded at CPz) are at the bottom. Black lines are for rare events, grey lines for frequent ones. The thin lines are intended to illustrate the direct comparison between *frequent* and *rare* predictions in the prediction-C task.

Error rates and mean RTs, averaged across participants, are displayed in the upper panels of Figure 2 and are compiled in Table 1. ANOVA results are summarized in Table 2. As expected, there were large oddball effects: more errors were committed, *F*(1, 19) = 151.2, *p* < .001, and responses were slower for rare than frequent events, *F*(1, 19) = 125.4, *p* < .001. All effects of the Task/Prediction factor were significant and were, therefore, analyzed in subsets of the data.

**Table 1.** Means Across Participants

	standard oddball	prediction: frequent	prediction: rare
RESPONSE TIMES (ms)			
frequent stimuli	320 (59)	359 (63)	408 (68)
rare stimuli	429 (53)	546 (85)	450 (120)
ERROR RATES (%)			
frequent stimuli	2.0 (2.1)	1.0 (0.9)	3.9 (5.4)
rare stimuli	32.9 (16.7)	29.2 (13.1)	10.5 (11.5)
P300-500 (CPz) (ěV)			
frequent stimuli	5.7 (4.7)	12.7 (6.8)	20.6 (8.6)
rare stimuli	21.9 (7.2)	24.4 (8.1)	25.8 (10.3)
P300-700 (CPz) (ěV)			
frequent stimuli	3.1 (4.6)	9.7 (5.8)	17.7 (7.4)
rare stimuli	15.3 (6.0)	20.9 (7.0)	22.4 (9.7)

*Note*. Interindividual standard deviations in brackets. Measurement units of the entered numbers (ms, %, μV) are indicated in the left column.

**Table 2.** Results of ANOVA *F*- and *p*-values on Error Rates and Response Times

	overall	*frequent* vs. *rare* predictions	oddball vs. *frequent* predictions	oddball vs. *rare* predictions
ERROR RATES			
Event Frequency	**151.2**	76.8	152.4	72.9
	<.001	<.001	<.001	<.001
Task & Prediction	14.3	**42.6**	**1.0**	**19.8**
	<.001	<.001		<.001
EF × T&P	17.9	**32.9**	**0.3**	**25.6**
	<.001	.004		<.001
RESPONSE TIMES			
Event Frequency	**125.4**	63.8	365.3	31.9
	<.001	<.001	<.001	<.001
Task & Prediction	13.3	**5.6**	**23.1**	**8.2**
	<.001	.03	<.001	.01
EF × T&P	18.3	**28.6**	**12.9**	**8.8**
	<.001	<.001	.002	.008

*Note*. Error rates - upper half, response times - lower half. The overall ANOVA (left column) was conducted on the three-level factor Task & Prediction. ANOVAs on each pair of these three levels are presented in the three right columns. *F*-values reported in the text are printed in bold. *p*-values would be entered when *p* ≤.10 (here actually *p* ≤.03). EF abbreviates the Event Frequency factor and T&P the Task & Prediction factor.

*ANOVA of prediction task*: This ANOVA was conducted on the *frequent predicted* and *rare predicted* values for frequent and rare stimuli (left and right values in the Figure 2 panels, connected by thin lines).

*Error rates*. There were overall more errors after *frequent* than *rare* predictions, *F*(1, 19) = 42.6, *p* < .001, but, as indicated by the interaction of Prediction × Event Frequency: *F*(1, 19) = 32.9, *p* < .001, this was true for rare events only (29% errors after *frequent* predictions, 11% after rare ones, *F*[1, 19] = 40.7, *p* < .001, in separate analysis of rare events) whereas the reverse was true for frequent events (1% errors after *frequent*, 4% after *rare* predictions, *F*[1, 19] = 6.0, *p* = .02). Thus, more errors were committed after incorrect than correct predictions, particularly for rare events (18% difference) but also for frequent ones (3% difference). The Prediction × Event Frequency interaction also indicated that oddball effects were larger after *frequent* than *rare* predictions: Rare events elicited 28% more errors than frequent events after *frequent* predictions, *F*(1, 19) = 96.4, *p* < .001, and 7% more errors after *rare* predictions, *F*(1, 19) = 6.4, *p* = .02.

*Response Times*: Responses were overall slower after *frequent* than *rare* predictions, *F*(1, 19) = 5.6, *p* = .03, but, as indicated by the Prediction × Event Frequency interaction: *F*(1, 19) = 28.6, *p* < .001, this was true with rare events only (546 ms vs. 450 ms; *F*[1, 19] = 21.2, *p* < .001) whereas responses to frequent events were slower after rare than after *frequent* predictions (408 ms vs. 360 ms; *F*[1, 19] = 18.6, *p* < .001). Thus, responses were slower after incorrect than correct predictions, more so for rare than for frequent stimuli (96 ms vs. 48 ms delay). The Prediction × Event Frequency interaction also reflected that oddball effects were much larger after frequent than after *rare* predictions: Responses were slower to rare than to frequent events by 186 ms after *frequent* predictions, *F*(1, 19) = 138.5, *p* < .001, and by 42 ms after *rare* predictions, *F*(1, 19) = 3.4, *p* = .08, not significant.

*Comparisons between standard oddball and prediction tasks*: Data from the standard oddball task were compared to either frequent or *rare* predictions from the prediction-C task—that is, in Figure 2 the middle values were compared to either the left or right values.

*Error rates*. Error rates did not differ between the standard oddball task and *frequent* predictions, (Task and Task × Event Frequency: *F*[1, 19] ≤ 1.0, *ns*). Compared to *rare* predictions, standard oddball error rates did differ (Task: *F*[1, 19] = 19.8, *p* < .001; Task × Event Frequency: *F*[1, 19] = 25.6, *p* < .001) being much higher for rare events (33% vs. 11%; *F*[1, 19] = 24.5, p < .001) in contrast to frequent events (2% vs. 4%; *F*[1, 19] = 2.2, *p* = .16, *ns*).

*Response Times*: Standard oddball RTs differed both from *frequent* predictions (Task: *F*[1, 19] = 23.1, *p* < .001; Task × Event Frequency: *F*[1, 19] = 12.9, *p* = .002) and from *rare* predictions (Task: *F*[1, 19] = 8.2, *p* = .01; Task × Event Frequency: *F*[1, 19] = 8.8, *p* = .008). Separate analyses of frequent and rare events showed that for rare events RTs were as fast in standard oddball as after *rare* predictions (430 ms vs. 450 ms), *F*(1, 19) = 0.7, not significant, thus faster than after *frequent* predictions (546 ms), *F*(1, 19) = 32.2, *p* < .001, and for frequent events RTs were even faster in standard oddball than after *frequent* predictions (320 ms vs. 360 ms), *F*(1, 19) = 4.7, *p* = .04, and markedly faster than after *rare* predictions (408 ms), *F*(1, 19) = 26.4, *p* < .001. Thus, RTs in the standard oddball task were at least as fast as for correctly predicted stimuli, both frequent and rare. Thereby, the oddball effect (rare-frequent stimuli) of 110 ms in the standard oddball task lay in-between the oddball effects for *frequent* and *rare* predictions (186 ms and 42 ms).

#### Prediction probabilities

In the prediction-C task, participants made *rare* predictions in 20.1% of trials. Rare stimuli were actually presented in 19.6% of trials. These subjective and objective probabilities did not differ from each other, *F*(1, 19) = 0.2, not significant.

### P3 component

**Figure 3.**
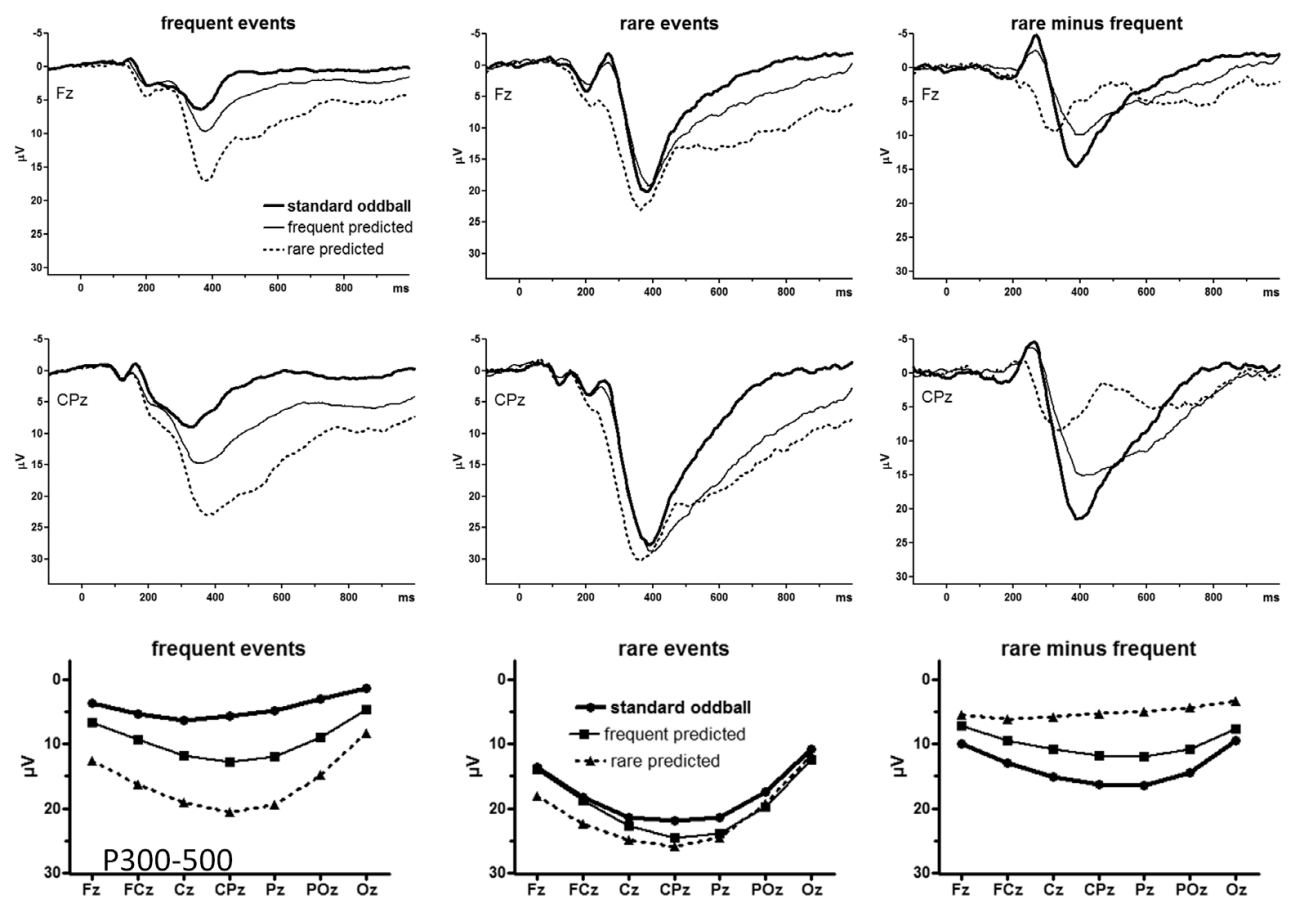
Grand average ERPs of the standard oddball task and the prediction-C task evoked by the imperative (to be predicted) letter. Bold lines are from the standard oddball task, thin solid lines from the prediction-C task when *frequent* was predicted, and dashed lines from the prediction-C task when *rare* was predicted. Left panels show the waveforms for frequent events, middle panels for rare events, and right panels the oddball effect (rare minus frequent). Upper panels show recordings from Fz, middle panels from CPz (either one referred to the nose). Negative voltage at these sites is plotted upwards. Time-point zero is onset of the imperative letter. The lower panels show mean amplitudes of P3 (300-500 ms after stimulus onset) at the seven midline recording sites. Negative is plotted upwards, for compatibility with the waveform graphs.

As evident in the grand average ERP waveforms (Figure 3) the P3 complex consisted of a large peak and a SW. It is doubtful whether the SW is a component of its own, following P3, or whether peak and SW should be treated as one component. Our main analysis will be restricted to the peak epoch, measured as mean amplitudes at 300-500 ms. Topographic profiles of these amplitudes at midline sites are displayed in the lower panels of Figure 3. CPz values are additionally depicted in Figure 2 (lower left panel) and compiled in Table 1.

#### P3 peak (P300-500)

ANOVA results are compiled in Table 3. The omnibus ANOVA confirmed the visual impressions from Figure 3 that amplitudes were largest at CPz, Cz, and Pz (Recording Site: *F*[6, 114] = 47.5, *p* < .001), were larger for rare than frequent events (Event Frequency: *F*[1, 19] = 120.4, *p* < .001) and that this oddball effect was largest at CPz and Pz (Recording Site × Event Frequency: *F*[6, 114] = 20.5, *p* < .001). Importantly, the main effect and all interactions of the Task/Prediction factor were significant and were, therefore, further analyzed in ANOVAs on subsets of the data. Main effects of Event Frequency and Recording Site and their interaction will not be again reported.

*ANOVA on prediction task*. The oddball effect was larger after frequent than after *rare* predictions (Prediction × Event Frequency: *F*[1, 19] = 10.6, *p* = .004; Event Frequency after *frequent* predictions: *F*[1, 19] = 132.8, *p* < .001, after *rare* predictions, *F*[1, 19] = 11.5, *p* = .003). Moreover, as indicated by the interaction of Prediction × Event Frequency × Recording Site: *F*(6, 114) = 9.3, *p* < .001, and illustrated in the lower right panel of Figure 3, the oddball effect after *frequent* predictions was largest at CPz and Pz, as may be expected for P3b (Event Frequency × Recording Site separately for *frequent* predictions, *F*[6, 114] = 14.5, *p* < .001), but was flat after *rare* predictions, *F*(6, 114) = 2.7, *p* = .06, not significant. When the Prediction × Event Frequency interaction was resolved to effects of Prediction separately for frequent and rare events (there was additionally a main effect of Prediction in the overall ANOVA, *F*[1, 19] = 20.4, *p* < .001) P3 amplitudes were larger after rare (incorrect) than frequent (correct) predictions for frequent events (Prediction: *F*[1, 19] = 78.5, *p* < .001) whereas the large P3s evoked by rare events did not differ between rare (correct) and frequent (incorrect) predictions (Prediction: *F*[1, 19] = 1.1, *ns*). Actually, there was some effect of Prediction with rare events at anterior sites Fz and FCz, probably reflecting an overlapping anterior feedback-related negativity with incorrectly predicted rare events. To detail, the interactions of Prediction × Recording Site: *F*(6, 114) = 18.3, *p* < .001, and Prediction × Event Frequency × Recording Site: *F*(6, 114) = 9.3, *p* < .001, prompted separate analyses of the Prediction effect at each recording site for frequent and rare events. With frequent events (Prediction × Recording Site: *F*[6, 114] = 15.5, *p* < .001) P3 was larger after incorrect than correct predictions at each site, *F*(1, 19) ≤ 83.8 and ≥ 20.6, *p* < .001, throughout, with largest mean differences at CPz, Pz, Cz, as expected for the P3b component. In contrast, with rare events (Prediction × Recording Site: *F*[6, 114] = 13.3, *p* < .001) P3 was smaller after incorrect than correct predictions at anterior sites (Fz: *F*[1, 19] = 10.9, *p* = .004; FCz: *F*[1, 19] = 6.6, *p* = .02; at other sites *F*[1, 19] ≤ 1.7, *p* ≥ .21).

**Table 3.** Results of ANOVA *F*- and *p*-Values on Error Rates and Response Times

	overall	*frequent* vs. *rare* predictions	oddball vs. *frequent* predictions	oddball vs. *rare* predictions
Recording Site	**47.5**	46.1	44.4	46.5
	<.001	<.001	<.001	<.001
Event Frequency	**120.4**	61.0	176.5	80.6
	<.001	<.001	<.001	<.001
RS × EF	**20.5**	7.2	33.8	17.0
	<.001	.002	<.001	<.001
Task & Prediction	20.8	**20.4**	**11.2**	**25.5**
	<.001	<.001	.003	<.001
RS × T&P	12.3	**18.3**	**9.8**	**11.4**
	<.001	<.001	<.001	<.001
EF × T&P	17.2	**10.6**	**11.5**	**24.6**
	<.001	.004	.003	<.001
RS × EF × T&P	11.3	**9.3**	**4.2**	**17.7**
	<.001	<.001	.02	<.001

Note. Mean amplitudes 300-500 ms after stimulus onset. The overall ANOVA (left column) was conducted on the three-level factor Task & Prediction. ANOVAs on each pair of these three levels are presented in the three right columns. *F*-values reported in the text are printed in bold. RS = Recording Site factor, EF = Event Frequency factor, and T&P = Task & Prediction factor.

*Comparisons between standard oddball and prediction tasks*. P3 amplitudes were smaller throughout in the standard oddball task than in prediction-C condition, both when frequent and when rare was predicted (Task: *F*[1, 19] ≥ 11.2, *p* ≤ .003). Differences were largest at CPz and Pz (Task × Recording Site: *F*[6, 114] ≥ 9.8, *p* < .001) and occurred mainly with frequent stimuli, as reflected by the Task × Event Frequency interaction: *F*(1, 19) ≥ 11.5, *p* ≤ .003, and by effects of Task being significant in subsequent separate analyses for frequent stimuli, *F*(1, 19) ≥ 24.8, *p* < .001, but not for rare stimuli, *F*(1, 19) ≤ 2.8, not significant. This increase of P3 for frequent events in the prediction-C task reduced the difference between rare and frequent events in this task. Therefore, the Task × Event Frequency interactions also meant that oddball effects (rare vs. frequent) were larger in the standard oddball than in prediction-C task. Differences were largest at CPz and Pz (lower right panel of Figure 3). The pertinent interaction of Event Frequency × Recording Site × Task was of moderate size when comparing standard oddball with *frequent* predictions where the oddball effect was smaller but had similar topography, *F*(6, 114) = 4.2, *p* = .02, and was large when comparing standard oddball with *rare* predictions where the oddball effect was topographically flat, *F*(6, 114) = 17.7, *p* < .001.

#### P3 peak (P300-500) versus P3 peak & slow wave (P300-700)

As noted above, Figure 3 suggests that the P3 complex consisted of a large peak and a following SW. To clarify whether results would change when measurement of the P3 complex also includes the SW, P3 & SW was measured by averaging amplitudes across 300-700 ms after stimulus onset. This measure (see lower right panel of Figure 2) was directly compared to the P3 peak measure used so far (300-500 ms) in the same ANOVAs as before, with the additional factor Measure (P3 peak vs. P3 & SW). Effects of this factor will be reported only.

*ANOVA on prediction task*. P3 peak was generally larger than P3 & SW, *F*(1, 19) = 69.0, *p* < .001. Both measures were largest at CPz, and the interaction of Recording Site × Measure: *F*(1, 19) = 5.1, *p* = .02, above all indicated that the two measures differed most where amplitudes were large. Of most interest, all other interactions of Measure were not significant, all *F*s ≤ 1.4, not significant, for interactions including Prediction, all *F*s ≤ 3.9, all *p*s ≥ .06, for interactions including Frequency. It may be concluded that, in this prediction task, the SW behaved like P3 peak.

*Comparison between standard oddball and prediction task*. In these ANOVAs (on standard oddball vs. *frequent* predictions, and vs. *rare* predictions) the Measure factor, among other effects, yielded interactions of Measure × Task: *F*(1, 19) ≥ 9.1, *p* ≤ .007, Measure × Task × Event Frequency: *F*(1, 19) ≥ 20.2, *p* < .001, and Measure × Task × Event Frequency × Recording Site: *F*(6, 114) ≥ 7.5, *p* ≤ .001. This pattern reflected that the two measures differed more from each other in the standard oddball task than in the prediction task. Inspection of Figure 3 suggests that this occurred because P3 returned earlier to baseline in the standard oddball task such that the SW part was distinctly smaller than in the prediction task. Comparison of the lower left and lower right panels in Figure 2 suggests as major difference that the large P3 evoked by rare stimuli in standard oddball became smaller when measured from 300 ms until 700 ms.

Therefore, when ANOVAs on standard oddball versus prediction were conducted on P3 & SW as the only measure (leaving out P300-500), the major difference from the ANOVA on P3 peak, reported above, was that the main effect of Task increased further, from previously *F*(1, 19) = 11.2 and 25.5 in standard oddball versus *frequent* and versus *rare* predictions, to *F*(1, 19) = 17.9 and 35.1. Correspondingly the interaction of Event Frequency × Task decreased, from previously *F*(1, 19) = 11.5 and 24.6 to *F*(1, 19) = 0.2, not significant, and 13.1. This occurred because P3 & SW amplitudes were generally smaller in the standard oddball than in the prediction task, both for frequent and rare stimuli, whereas P3 peak had been smaller in standard oddball than in prediction for frequent stimuli only.

#### Predicting without confirming

**Figure 4.**
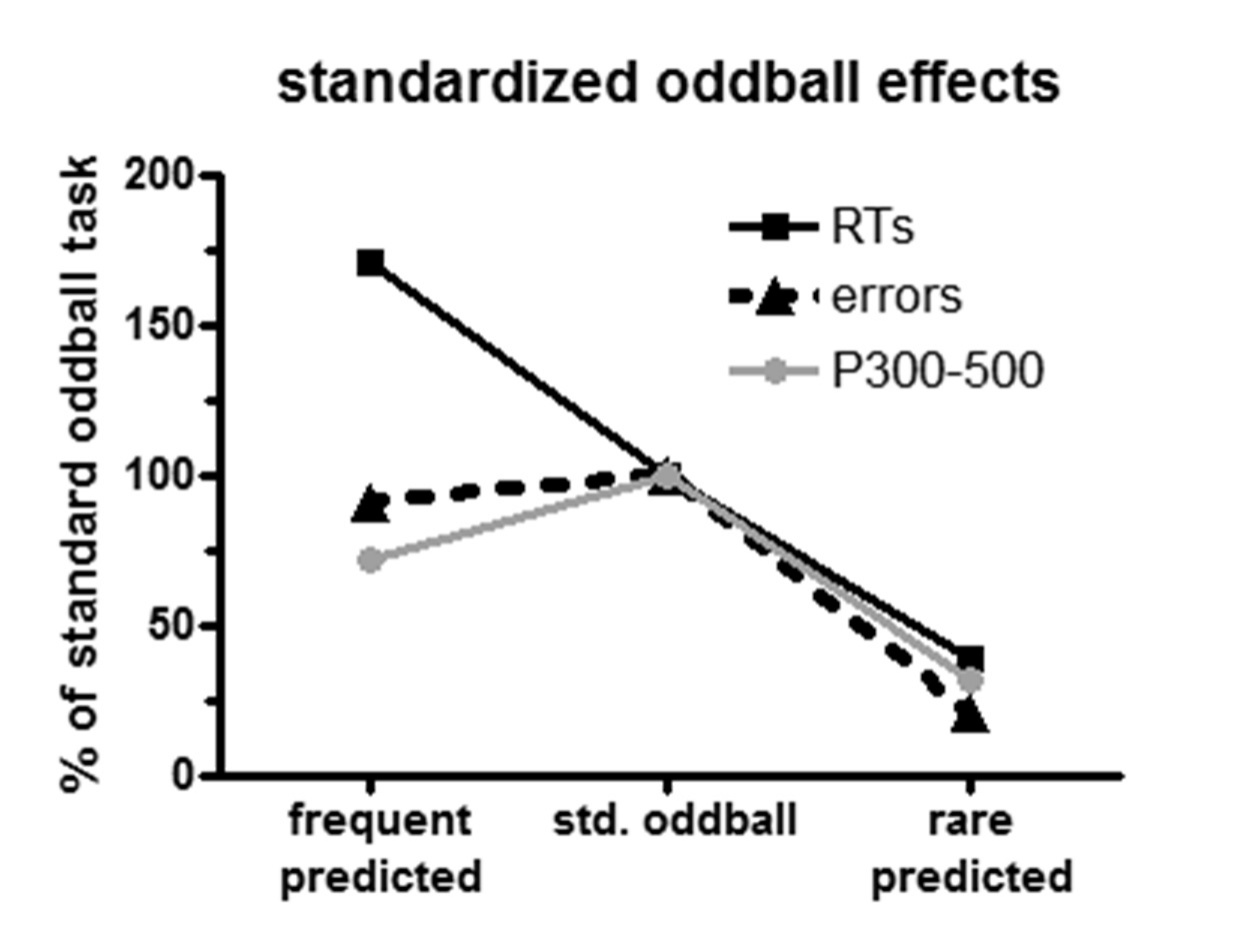
Grand average ERPs evoked in the prediction-C and the prediction-noC tasks by the letter that had to be predicted. Black lines, from prediction-C, are identical with Figure 3, grey lines are from prediction-noC. Solid lines are from trials when frequent was predicted, and dashed lines from trials when rare was predicted. Left panels show the waveforms for *frequent* events, middle panels for *rare* events, and right panels the oddball effect (rare minus frequent). Upper panels show recordings from Fz, lower panels from CPz (either one referred to the nose). Negative voltage at these sites is plotted upwards. Time-point zero is onset of the letter that was to be predicted.

In order to clarify whether results of the prediction-C task were affected by the additional requirement of confirming the outcome by keypress, P300-500 amplitudes were compared between the prediction-C and prediction-noC tasks, in ANOVAs where the factor Confirmation (yes, no) was added to the Prediction, Frequency, and Recording Site factors. Effects of the Confirmation factor will be reported only. Grand mean waveforms are displayed in Figure 4 (with the black waveforms identical to Figure 3).

 P3 peaks were larger in the prediction-C than in the prediction-noC task (Confirmation, *F*[1, 19] = 5.4, *p* = .03) specifically at Fz, FCz, Cz, CPz, Confirmation × Recording Site: *F*(6, 114) = 10.2, *p* < .001. Importantly, effects of Confirmation did neither modify effects of Prediction nor of Event Frequency, all interactions *F*s ≤ 1.1, not significant. We conclude that the motor response added an anteriorly focused positive potential to P3, with similar amplitude in all conditions. Most probably, this is a response-related positivity distinct from P3 (Ouyang, Sommer, & Zhou, 2015; Verleger et al., 2014). 

### Summary of major results

**Figure 5.**
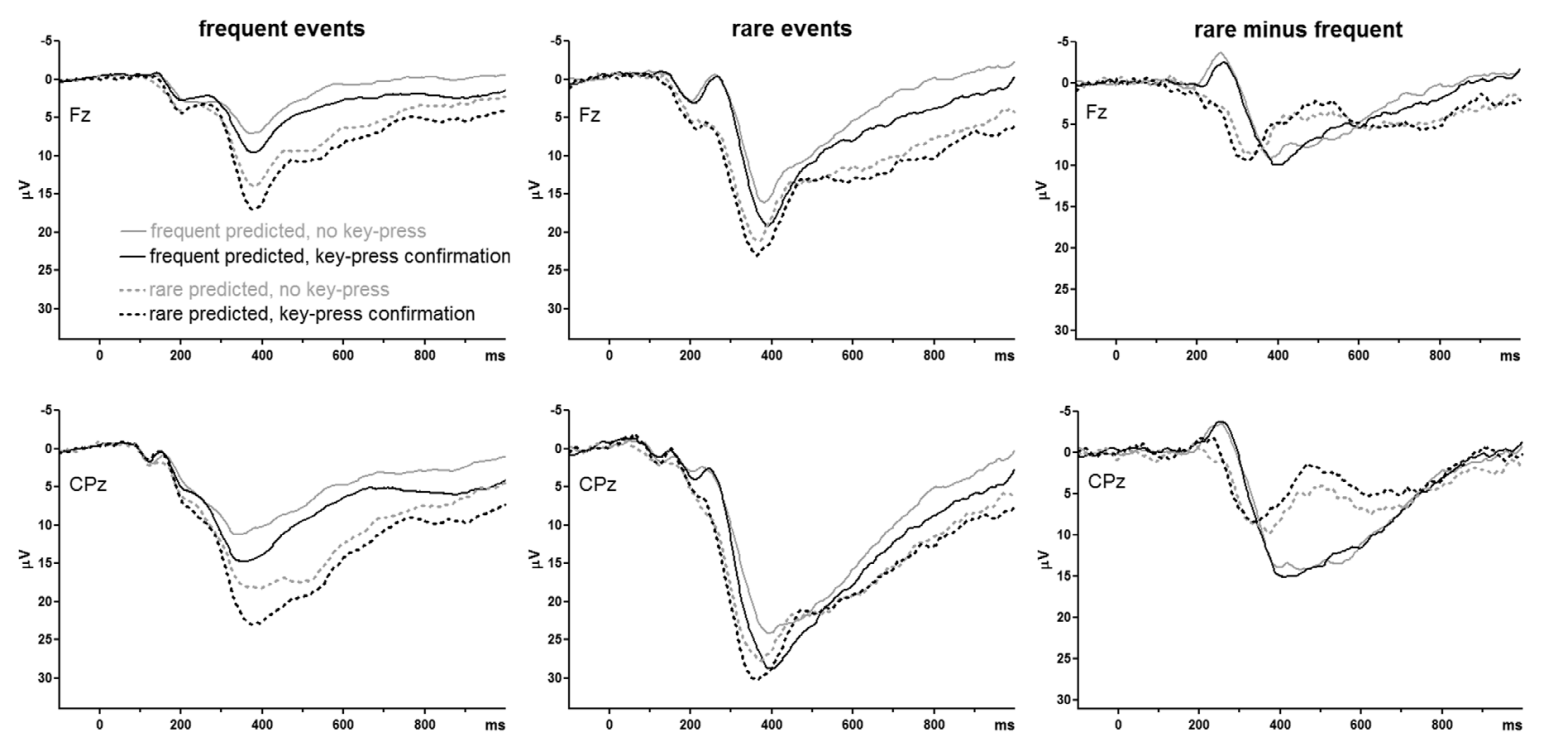
Summary view on oddball effects for RTRTs, error rates, and P3 amplitude (300-500 ms). To have comparable scales, oddball effects (i.e., the differences between rare and frequent stimuli) were set to 100% for the standard oddball task, and results from the prediction task are expressed as percentages relative to the standard oddball task.

Summary information on the variation of the oddball effect is displayed in Figure 5. To provide a common scale, oddball effects of the standard oddball task were set to 100%. As the figure shows, oddball effects decreased markedly and by similar extents for errors, RTs, and P3 when rare stimuli were predicted. In contrast, the three measures diverged when frequent stimuli were predicted: The oddball effect was similar to the standard oddball task for error rates, was increased for RTs, and was somewhat reduced for P3.

## Discussion

This study investigated whether behavior and P3 amplitudes in the oddball task reflect expectancy of frequent or of rare stimuli and in particular whether the oddball effect on P3 occurs because rare stimuli are unexpected. This was done by comparing results of the standard oddball task with results from a prediction task. In both tasks, alternative key-press responses were required to frequent and rare stimuli such that not only P3 amplitudes, but also RTs and error rates could be compared between tasks. There were clear oddball effects in both tasks: P3 amplitudes were larger, RTs were slower, and more errors were committed with rare than with frequent stimuli.

### Differences between *frequent* and *rare* predictions in the prediction task

 Hypothesis (2) said that the oddball effect in the prediction task would be smaller after rare than after *frequent* predictions. Indeed (Figure 5) this was true for RTs, error rates, and P3 amplitudes. The P3 results replicate our previous study (Verleger, Asanowicz, et al., 2015)^1^) and an early report by Tueting, Sutton, and Zubin (1970)^2^. However, we assumed that the reason for this decreased oddball effect is that, by having made this prediction, participants will be prepared to perceive and process rare stimuli. Thus, responses to rare stimuli, above all, were assumed to change after rare predictions. Indeed this applied to RTs and errors (Figure 2) where effects of prediction were larger for rare than for frequent stimuli. However, this did not apply to P3 amplitudes (Figures 2, 3). Rather, P3 amplitudes were reliably affected by expectancies only when stimuli were frequent, being larger with incorrectly than correctly predicted frequent stimuli. In contrast, the large P3 amplitudes for rare events were not affected by expectancies. The slight increase with correctly predicted rare stimuli, significant at Fz and FCz only, appears as a protracted effect of the large N2 in case of unpredicted rare stimuli, reducing the positive level of the following overlapping P3. (See upper middle panel of Figure 3). Thus, the assumption that P3 is increased when stimuli are unexpected was confirmed in the prediction task for frequent stimuli, but not for rare stimuli. This result lends itself to the interpretation that the process reflected by P3 was elicited in any case by rare stimuli and, therefore, could not become additionally increased when stimuli were expected or unexpected. 

### Similarities between the standard oddball task and either frequent or rare predictions

 Of most interest was whether the oddball effect after *frequent* or after *rare* predictions (hypotheses [3a] and [3b]) would more resemble the oddball effect from the standard oddball task. Results summarized in Figure 5 suggest that the oddball effects on P3 amplitudes and on error rates in the standard oddball task behaved similarly to *frequent* predictions, in line with hypothesis (3a). In contrast, effects on RTs in the standard oddball task lay in-between the values for *frequent* and *rare* predictions, in line with hypothesis (3c). To detail, the results for error rates were straightforward: These rates were as high with rare stimuli and as low with frequent stimuli as when frequent stimuli were predicted (Figure 2). This would suggest that participants expected frequent stimuli and prepared for frequent responses (cf. Miller, 1998) in the standard oddball task. Of course, this appears as a rational strategy because most stimuli were, by definition, frequent. Interestingly, however, RTs did not follow this logic but rather were as fast with rare stimuli as when rare stimuli were predicted and were even faster with frequent stimuli than when frequent stimuli were predicted (Figure 2). Such benefit-benefit patterns may be explained by simultaneous priming of both responses (Sommer et al., 1999). 

 The divergence between effects of expectancies on error rates and on RTs may indicate that processing occurs at various levels. Simultaneous priming of both responses may occur at the motor level where participants might prepare to respond quickly with either hand. This motor level may differ from the decision level which, as reflected in error rates, was biased towards triggering the frequent response. Unspecific priming of both responses at the motor level may later require inhibition of the motor cortex that would generate the incorrect response. Evidence in favor of this notion has been provided by transcranial magnetic probe stimulations (e.g., Leocani, Cohen, Wassermann, Ikoma, & Hallett, 2000; Verleger, Kuniecki, Möller, Fritzmannova, & Siebner, 2009) and by current-source density computations of EEG recordings (e.g., Praamstra & Seiss, 2005; Vidal, Burle, Grapperon, & Hasbroucq, 2011). 

 Of most interest was the oddball effect on P3 amplitudes. Like error rates, this effect closely resembled the values when frequent stimuli were predicted (Figure 5). However, this P3 effect is more difficult to interpret than the effect on error rates because it was due to changes in P3 evoked by frequent stimuli. This sensitivity to predictions on frequent stimuli is in contrast to effects on the fronto-central N2 component, well visible in Figure 3: Differences between *frequent* and *rare* predictions in the oddball effect on N2 (right panels of Figure 3) indeed were due to differences between predictions on rare stimuli (middle panels) rather than to changes with frequent stimuli (left panels). This is similar to results reported by Fogelson, Fernandez-del-Olmo, and Santos-Garcia (2011) where P3 amplitudes did not differ between predictable and unpredictable targets, but N2 amplitudes were increased when targets were unpredictable. Thus, it may be concluded that the N2 effect in the standard oddball task indicates that rare stimuli are unexpected, in contrast to the P3 effect. 

### Global differences between prediction and standard oddball tasks

Comparison of the P3 effects between the two tasks is impaired by the differences in time-courses of the P3 waveform. As inspection of Figure 3 suggests, the epoch of 300-500 ms, comprising P3’s peak, is indeed the relevant time segment in the standard oddball task. In contrast, in the prediction task P3 was embedded in a slow positivity that lasted until 700 ms and later. Complicating this issue, the relation of P3 peak and SW differed between frequent and rare stimuli within the prediction task. With frequent stimuli, effects on P3 and SW were indistinguishable: The SW appeared to be an integral part of the P3 complex. With rare stimuli, the SW did not increase P3’s peak but followed it. Thereby, comparison of P3 evoked by rare stimuli between prediction and standard oddball tasks depended on how P3 was measured. P3 amplitudes did not differ between tasks for rare stimuli with the 300-500 ms measure (except for some overlapping negativity at anterior sites) but were clearly larger in the prediction than in the standard oddball task when the SW was included by using the 300-700 ms measure. The following discussion is based on this latter perspective, mainly because the SW was so obviously a genuine part of the P3 complex with frequent stimuli that excluding it for rare stimuli seems arbitrary. Thus, P3 amplitudes were smaller throughout the standard oddball task than the prediction task for frequent events and at least the later SW part of P3 was also smaller for rare events (Figures 2, 3). It might be suspected that this increase in the prediction task is related to the unusual requirement of confirming the stimuli by key-press in this task (see Introduction). However, analysis of the prediction-noC task (Figure 4) showed that this did not account for the major part of the difference.

 An obvious account of these global differences between tasks relates to what was intended in presenting these tasks: Explicit predictions were made about the stimuli in the prediction task but not in the oddball task. Thereby, the stimuli might have attained more relevance in the prediction task than in the oddball task. Alternative accounts may relate to secondary differences between tasks as already mentioned in the Methods section. One obvious difference was the reward associated to correctly predicted stimuli of the prediction task, about which participants were constantly reminded by the feedback screens provided after every 20 trials. Clearly, this is a confound, but we considered this necessary to motivate participants for remaining involved in making predictions rather than just mechanically pressing some key. Another obvious difference between tasks was their difference in intervals between successive letters which amounted to about 1.2 s in the standard oddball task and about 3 s in the prediction task. Indeed, P3 amplitudes have been reported to increase with increasing interstimulus intervals (Gonsalvez et al., 1999; Steiner, Brennan, Gonsalvez, & Barry, 2013) above all with frequent stimuli, as was the case here, although effects may be weak and not always present (e.g., Polich, 1987, 1990; Polich & Bondurant, 1997). One might try and avoid this difference between standard oddball and prediction tasks in future studies by extending the interstimulus intervals of the standard oddball task. However, this would introduce another difference because in the present version the P3-evoking stimuli were equally preceded at 1.3-1.5 s by another event which was the previous stimulus in the standard oddball and the guess prompt in the prediction task. Moreover, extended intervals in the standard oddball task would not be typical of the 1 - 2 s intervals most frequently used in this task.^3^

### Interim summary

Oddball effects (rare-frequent differences) on RTs lay in the standard oddball task in-between effects after *frequent* and *rare* predictions in the prediction task, probably because both responses were primed. In contrast, standard-oddball effects on P3 amplitudes and error rates closely resembled oddball effects after *frequent* predictions. This corroborates the notion that these effects on P3 occur because frequent stimuli are expected and rare stimuli are unexpected. However, this notion was put into doubt by a closer look at the results from the prediction task because, in this task, the modifications of oddball effects on P3 by expectancies were entirely due to altered amplitudes with frequent stimuli, whereas the large P3 amplitudes evoked by rare stimuli were insensitive to predictions (unlike RTs and error rates). Therefore, it cannot be said that large P3s with rare stimuli reflect unexpectedness of these stimuli. Thus, our attempt at accounting for the oddball effect on P3 in terms of expectancy has resulted in a dilemma.

### Theoretical accounts

**Figure 6.**
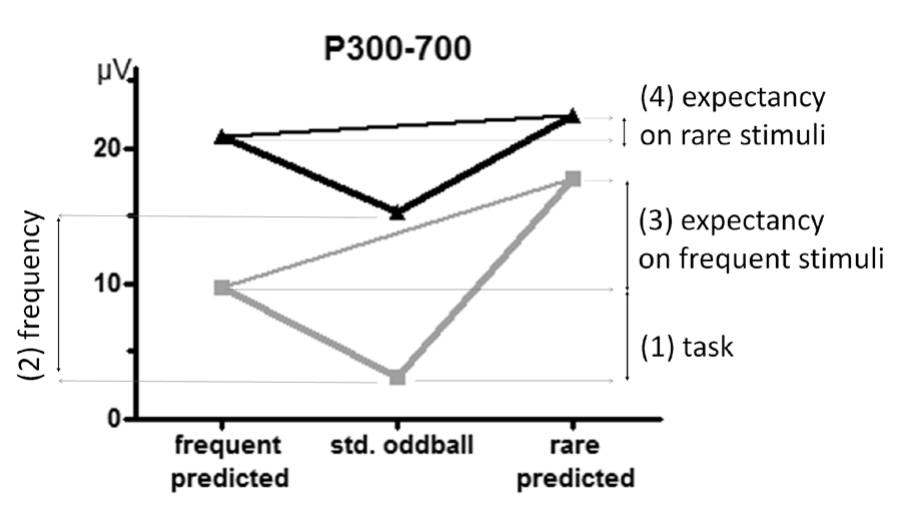
Overview of effects on the P3 complex. Data were taken from the lower right panel of Figure 2.

This dilemma made us take a look at current concepts and hypotheses about P3: How may the effects of expectancy, frequency, and task be accounted for? To summarize, the following effects on P3 were here obtained (cf. Figure 6): (1) The task effect: P3 increased from standard oddball to prediction. (2) The event-frequency (oddball) effect: P3 increased in all tasks from frequent to rare stimuli. (3) The effect of expectancy on frequent stimuli: P3 increased from *frequent*-predicted to *rare*-predicted trials. (4) The absent expectancy effect on rare stimuli: P3 did not increase from rare-predicted to frequent-predicted trials.

Table 4 summarizes how several intervening variables and underlying processes can deal with this pattern of effects. Table entries will be described and discussed in the following.

**Table 4.** Performance of Several Constructs and Hypotheses About P3 in Accounting for the Present Results

	(1) task	(2) frequency	(3) expectancy on frequent stimuli	(4) no expectancyon rare stimuli
**stimulus attributes**				
unexpected	0	(+)	+	-
awaited	0	-	-	0
primed S	+	+	0	+
primed S & R	+	+	+	-
relevant	+	+	+	+
**processes**				
inhibition for focusing attention	0	0	0	0
inhibiting primed responses	0	+	+	0
memory storage	+	0	0	0
context updating	+	0	0	+
closure / network reset	+	0	-	0
response facilitation	0	0	0	0
decision	-	+	0	0
reactivating S-R links	+	+	+	-
generating conscious representations	0	0	0	+

*Note*. „+” is entered for results compatible with a given concept, „0” means that the concept does not provide an account for the result, and „-” means that the result was opposite to what the concept implies.

As discussed before, the notion that P3 is evoked by unexpected stimuli may, of course, easily account for the P3 increase with unexpected frequent stimuli (3). However, it cannot account for the absent expectancy effect with rare stimuli (4) and, therefore, has problems in accounting for the oddball effect (2) even though rare stimuli are unexpected in the oddball task. In addition, the task effect does not lend itself to an easy interpretation: Why should stimuli, both correctly and incorrectly predicted ones, be more unexpected in the prediction task than in the standard oddball task (1)?

Mirror-symmetric arguments apply to the notion that P3 is evoked by awaited stimuli: The non-significant increase with predicted rare stimuli (4) would have fitted well but not being significant and, moreover, probably being due to overlap of anterior negativity, does not do so. Moreover, the effect of expectancy on frequent stimuli (3) clearly conflicts with this notion, and the task effect (1) cannot be accounted for. Most importantly, the P3 increase with rare stimuli (frequency effect [2]) behaved in the standard oddball task like the frequency effect in the prediction task when frequent, rather than rare, stimuli were predicted, opposite to what this notion assumes.

 Kahneman and Tversky’s (1982) proposal that P3 increases when primed dispositions about stimuli are violated (for a similar suggestion see Gonsalvez et al.’s, 1999, template-updating hypothesis) may account for the task effect (1) because there were longer intervals between stimuli in the prediction than in the standard oddball task, thus primed dispositions might have decayed more. Importantly, however, the expectancy effect (3) cannot be readily explained by primed dispositions: Why would priming depend on conscious expectancies (cf. the opposite effects of expectancy and priming on P3 and Mismatch Negativity described by Ritter et al., 1999)? Being at the heart of the primed disposition notion, the frequency effect (2) can be easily accounted for, as can the absence of the expectancy effect with rare stimuli (4). 

 The notion of primed dispositions may be extended to include dispositions about responses, in particular dispositions about S-R links (Verleger, Metzner, Ouyang, Śmigasiewicz, & Zhou, 2014; similarly Steiner et al., 2013). This extended conception of primed dispositions may account for the task (1) and frequency effects (2) in the same way as the notion of dispositions about stimuli. This extended predisposition view may even account for the expectancy effect (3) because predictions on stimuli were made by pressing their associated keys. Thus, pressing some key may prime the associated stimulus, such that presentation of the alternative stimulus may violate this primed predisposition (cf. the “event file” conception suggested by Hommel, Müsseler, Aschersleben, & Prinz, 2001; e.g., more recently Kühn, Keizer, Colzato, Rombouts, & Hommel, 2011). On the other hand, this rationale should as well apply to rare stimuli, so the absent expectancy effect with rare stimuli (4) runs counter to this account. 

 Stimulus relevance has often been suggested as a factor affecting P3, for instance, by Begleiter, Porjesz, Chou, and Aunon (1983) , and Johnson (1986; speaking of “stimulus value” or “significance”) as well as in the context of other concepts discussed below. Indeed, this variable might account for all these effects, as follows. The task effect (1) reflects that stimuli have more relevance in the prediction task than in the standard oddball task: Each single stimulus had to be predicted, then confirms or disconfirms that prediction and, moreover, may yield some reward for correct guessing. Moreover, the increased interstimulus intervals alone may boost relevance of single stimuli. That the effect was restricted here and in previous cases (Polich, 1987, 1990; Polich & Bondurant, 1997) to frequent stimuli may have happened because rare stimuli are relevant in any case, as follows. The oddball effect (2) may reflect that rare stimuli are more relevant, by interrupting routine behavior and requiring non-routine responses. The expectancy effect on frequent stimuli (3) (larger P3 after rare than *frequent* predictions) may occur because making the rare prediction lends additional value to the trial for whatever stimulus may come, so the same frequent stimulus will have more relevance when showing up after the rare prediction. This may not have anything to do with the fact that these stimuli are unexpected. Finally, the lacking expectancy effect with incorrectly versus correctly predicted rare stimuli (4) may be due to the fact that rare stimuli are relevant in this task in any case. 

Thus, stimulus relevance may be the common factor mediating all these effects. What, then, is the underlying process reflected by P3?

 According to Polich (2007, 2012) P300 reflects a chain of two processes: an inhibitory process to enhance the attentional focus on relevant stimulation, followed by memory storage. With respect to inhibition, it appears unclear why ongoing processing or distracting information would be more inhibited in the prediction task than in the standard oddball task (effect [1]). The need for inhibition seems more plausible when stimuli are rare (2) and unexpected (3). What has to be inhibited in these cases is the primed frequent response (see separate entry in Table 4). But when the inhibition concept is restricted to processes affecting stimulus classification (which appears to be the view of Polich, 2007, 2007, 2012) it remains unclear why inhibition would increase with unexpected or rare stimuli. Accordingly, the inhibition notion also remains neutral to the absent expectancy effect with rare stimuli (4). 

 With respect to memory storage, it makes sense to assume that stimuli are stored more intensively in the prediction task (1) because participants might base their future predictions on the present outcomes (Munson et al., 1984) whereas there is hardly any need for such storage in the standard oddball task. However, accounts of this notion for the frequency (2) and expectancy (3) effects remain ambiguous (therefore are coded “0” in Table 4). For brevity, we will mention counterarguments only. Storing the rare outcome more intensively than the frequent one (2) may indeed make sense in the prediction task because this may be relevant for choosing the next predictions, but it is not clear why this should occur in the standard oddball task, too. Thus, the frequency effect should be larger in the prediction task. It is likewise unclear why unexpected stimuli should be stored more intensively than expected ones (3) in view of century-long evidence for learning by success rather than by failure. Therefore, the memory storage notion would be more compatible with larger P3s by expected stimuli, but this effect was not significant (4). 

 According to Donchin (1981), P3 reflects context updating, meaning that some model of the environment (“schema”) is updated “when there is a conflict between new information and expectations derived from a ‘schema’” (Kamp, Brumback, & Donchin, 2013). This schema is assumed to be involved in the metacontrol setting of priorities, biases, and probabilities (Donchin & Coles, 1988). The task effect (1) makes much sense because the schema may be more sensitive to stimulus identity when stimuli have to be actively predicted. Problematic is the frequency effect (2). When properly updated, the model of the environment will certainly allow for the occurrence of both frequent and rare stimuli, so why should the schema be more updated when encountering a rare stimulus than a frequent one? Only if including one stimulus only, like a mismatch negativity process, the schema would require updating after rare stimuli. Such reduction of its applicability to isolated stimuli would deprive the context-updating hypothesis of its essential content, at least in Verleger’s (1988) view. The same applies to the effects of expectancy on frequent stimuli (3): A good model, properly adapted to the environment, would certainly allow for the fact that non-predicted stimuli may occur. If the model includes one stimulus only, then unpredicted stimuli might continuously require updating of the model which in this case would never reach proper understanding of what is going on. Therefore, the absence of the expectancy effects with rare stimuli (4) does fit the context-updating notion. 

 According to Desmedt and Debecker (1979) , P3 reflects the closure of cognitive epochs after decisions on relevant signals have been reached, resetting the brain’s processing system (cf. Bouret & Sara, 2005). P3 will be the larger, the longer some cognitive epoch had lasted and the more relevant the signal is.)^4^ Leaving the relevance notion aside which may account for all our effects (cf. above), closure can explain the task effect (1) by the longer interstimulus intervals, and the frequency effect (2) in the standard oddball task by assuming that cognitive epochs last from one rare (“target”) stimulus to the next and that, therefore, frequent stimuli do not close epochs (Desmedt & Debecker, 1979). However, the frequency effect should play a minor role in prediction tasks because it seems reasonable to assume that the major cognitive epoch in this task lasts from predicting the next stimulus to its occurrence, be it frequent or rare. Additional simultaneous presence of a long-ranging epoch may be postulated (Verleger, 1988), from one rare predicted stimulus to the next rare one, encompassing several short-range epochs from prediction to outcome, but when situations are so different between tasks then frequency effects should also differ between tasks. Moreover, when participants wait for some stimuli that fulfill criteria for closing the epoch, the closure notion cannot easily account for the facts that incorrectly predicted frequent stimuli evoked larger P3s than predicted ones (3) and that predicted rare stimuli did not evoke larger P3s than unpredicted ones (4). 

 According to Nieuwenhuis, Aston-Jones, and Cohen (2005) P3 reflects response facilitation after decisions on relevant signals have been reached. It is not easy to see how response facilitation may account for the task effect (1) because larger P3s than in standard oddball were not only obtained in the prediction-C task but as well in the prediction-noC task where no responses were required at all. This issue remains virulent with the effects of frequency (2), unexpectedness (3) and expectedness (4): When responses are required to the stimuli, it may indeed be argued that responses need facilitating with rare and unexpected stimuli. But the same effects were obtained in the prediction-noC task where no responses were required, so the notion of response facilitation cannot provide satisfactory accounts. 

 More recently, P3 has been considered to directly reflect the decision process rather than some post-decision adaptation (Kelly & O’Connell, 2013; O’Connell, Dockree, & Kelly, 2012). The task effect (1) may conflict with this notion: Why would P3 be larger with predicted stimuli that do not require a clear decision on action than in the standard oddball where such decision is required? In contrast, effects of frequency (2) are easily explained by the diffusion model underlying the decision conception because of asymmetry of thresholds for frequent and rare decisions relative to the starting point of the diffusion process that drives the decision (Twomey, Murphy, Kelly, & O’Connell, 2015). But effects of unexpectedness in the prediction task come as a challenge: What are the decisions reflected by P3? These might be the decisions “yes, I was right” and “no, I was wrong”. But why does the decision *wrong* produce a larger P3 than the decision *right* with frequent stimuli (3) and not with rare stimuli (4)? It appears that the model has to be further specified to deal with this situation. 

 Likewise recently, we endorsed the hypothesis of stimulus-response (S-R) link reactivation (e.g., Verleger et al., 2014 ; Verleger, Hamann, Asanowicz, & Śmigasiewicz, 2015). This hypothesis posits that a few fixed S-R links are established by instruction and practice (e.g., “stimulus A → left key”, “stimulus B → right key”). When, during some consecutive trials, only one of these S-R links was used, the other one will have to be reactivated when the corresponding stimulus is perceived, which is reflected by P3. It seems that this hypothesis simply postulates a mechanism for the effects of primed dispositions about S-R links discussed above. Thus, the task effect (1) is accounted for by lengthening of interstimulus intervals, the frequency effect (2) is easy to account for, as is the expectancy effect on frequent stimuli (3), but the absence of this effect with rare stimuli (4) is in conflict with this notion. 

 Finally, P3 has been suggested to reflect activation of some global workspace in producing consciousness (Dehaene, Sergent, & Changeux, 2003). This would imply that more conscious awareness is generated in the prediction task than in the standard oddball task (1), by rare than by frequent stimuli (2), by unexpected than expected frequent stimuli (3), and equal conscious awareness is generated by unexpected and expected rare stimuli (4). Undoubtedly, however, according to usual criteria all our stimuli were consciously perceived, and conscious awareness is considered to be an all-or-none phenomenon rather than a gradual one (Sergent, Baillet, & Dehaene, 2005) so it appears that this hypothesis can only account for the absent effect of expectancy (4). 

In conclusion, all discussed concepts have their problems in accounting for the effects obtained in the present study. Thus, while there appears to be no unequivocal disproof of the S-R link reactivation conception recently proposed by us, the most satisfactory account appears to be in terms of subjective relevance of stimuli. It may be speculated that the process reflected by P3 simply consists of assigning relevance to stimuli.

## Footnotes

2 Obtained from four participants, with recording sites at Fz and Cz only.

3 Another methodological concern relates to the order of the tasks. Indeed, order was balanced for the critical comparison between standard oddball and prediction-C tasks. However, when the standard oddball task preceded the prediction-C task it was still preceded by the prediction-noC task. So it might be suspected that the requirement to predict the upcoming stimuli always carried over to the standard oddball task, resulting in covert predictions in this task. Future studies should avoid this confound.

4 In arguing for the closure conception, Verleger (1988) worsened it, by combining it with the conviction held by many researchers at that time that P3 reflects “stimulus evaluation” only, independent of “response processing”. This “stimulus evaluation” notion has been disconfirmed by the bulk of empirical evidence, see Verleger (1997, 2010), with adverse implications for Verleger’s (1988) “closure of perceptual epochs”.
